# Pancreatic cancer accompanied by a moderate-sized pseudocyst with extrapancreatic growth

**DOI:** 10.1186/s12880-015-0055-2

**Published:** 2015-05-07

**Authors:** Yu Ohkura, Kazunari Sasaki, Masamichi Matsuda, Masaji Hashimoto, Takeshi Fujii, Goro Watanabe

**Affiliations:** Department of Gastroenterological Surgery, Hepato Pancreato Billiary Surgery Unit, Toranomon Hospital, 2-2-2 Toranomon, Minato-ku, Tokyo 105-8470 Japan; Department of Pathology, Toranomon Hospital, 2-2-2 Toranomon, Minato-ku, Tokyo 105-8470 Japan

**Keywords:** Slight pancreatic duct dilation, Extrapancreatic growth, Pancreatic adenocarcinoma, Pseudocyst

## Abstract

**Background:**

Pancreatic cancer accompanied by a moderate-sized pseudocyst with extrapancreatic growth is extremely rare. Diagnosis of pancreatic cancer on preoperative imaging is difficult when the pancreatic parenchyma is compressed by a pseudocyst and becomes unclear. Despite advances in imaging techniques, accurate preoperative diagnosis of cystic lesions of the pancreas remains difficult. In this case, it was challenging to diagnose pancreatic cancer preoperatively as we could not accurately assess the pancreatic parenchyma, which had been compressed by a moderate-sized cystic lesion with extrapancreatic growth.

**Case presentation:**

A 63-year-old woman underwent investigations for epigastric abdominal pain. She had no history of pancreatitis. Although we suspected pancreatic ductal carcinoma with a pancreatic cyst, there was no mass lesion or low-density area suggestive of pancreatic cancer. We did not immediately suspect pancreatic cancer, as development of a moderate-sized cyst with extrapancreatic growth is extremely rare and known tumor markers were not elevated. Therefore, we initially suspected that a massive benign cyst (mucinous cyst neoplasm, serous cyst neoplasm, or intraductal papillary mucinous neoplasm) resulted in stenosis of the main pancreatic duct. We were unable to reach a definitive diagnosis prior to the operation. We had planned a pancreaticoduodenectomy to reach a definitive diagnosis. However, we could not remove the tumor because of significant invasion of the surrounding tissue (portal vein, superior mesenteric vein, etc.). The fluid content of the cyst was serous, and aspiration cytology from the pancreatic cyst was Class III (no malignancy), but the surrounding white connective tissue samples were positive for pancreatic adenocarcinoma on pathological examination during surgery. We repeated imaging (CT, MRI, endoscopic ultrasound, etc.) postoperatively, but there were neither mass lesions nor a low-density area suggestive of pancreatic cancer. In retrospect, we think that the slight pancreatic duct dilation was the only finding suggestive of pancreatic cancer.

**Conclusions:**

It is difficult to diagnose pancreatic cancer with pseudocyst preoperatively. If a pancreatic cyst is found in patients who had normal tumor marker levels or no history of pancreatitis, we should always consider the possibility of pancreatic cancer. In such cases, slight pancreatic duct dilation may be a diagnostic clue.

## Background

Extrapancreatic growth of a moderate-sized pseudocyst is extremely rare. It is difficult to diagnose pancreatic cancer on preoperative imaging when the pancreatic parenchyma is compressed by a massive pseudocyst and becomes unclear on imaging. Despite the development and widespread use of imaging techniques, obtaining an accurate preoperative diagnosis of cystic lesions of the pancreas remains difficult. In this case, it was challenging to diagnose pancreatic cancer preoperatively as we were unable to accurately assess the pancreatic parenchyma, which had been compressed by a cystic lesion with extrapancreatic growth.

## Case presentation

**Figure 1:**
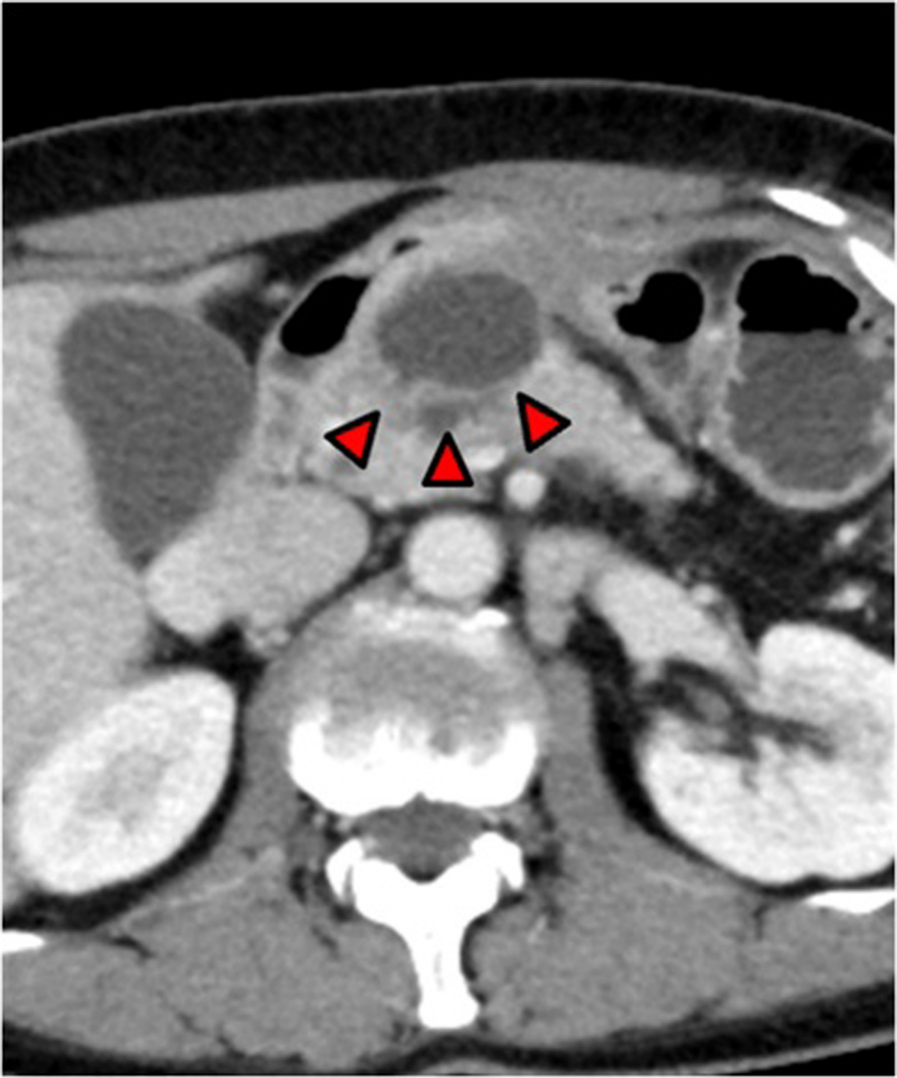
Enhanced CT scan. The cyst exhibited extrapancreatic growth with dilatation of the main pancreatic duct. The low-density area indicated by the arrows is the main pancreatic duct. There was neither a mass lesion nor a low-density area suggestive of pancreatic cancer.

**Figure 2:**
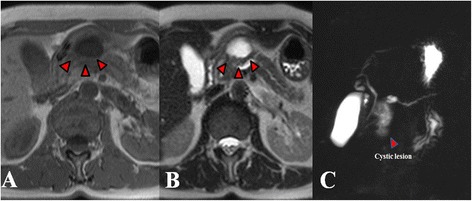
MRI. The massive simple cyst in the pancreatic body showed low signal intensity on **A)** T1-weighted MRI and **B)** high intensity on T2-weighted MRI. **C)** MRCP showed slight dilatation of the main pancreatic duct but stenosis of the duct of Wirsung was not seen.

**Figure 3:**
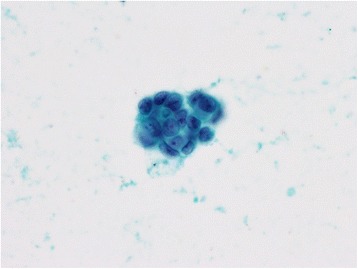
Cytology from the pancreatic cyst. Cytology from the pancreatic cyst revealed papillary cell clumps with nuclear expansion and clear nuclear bodies. The cells have irregular nuclei and a high level of chromatin content. Class IV (×60).

**Figure 4:**
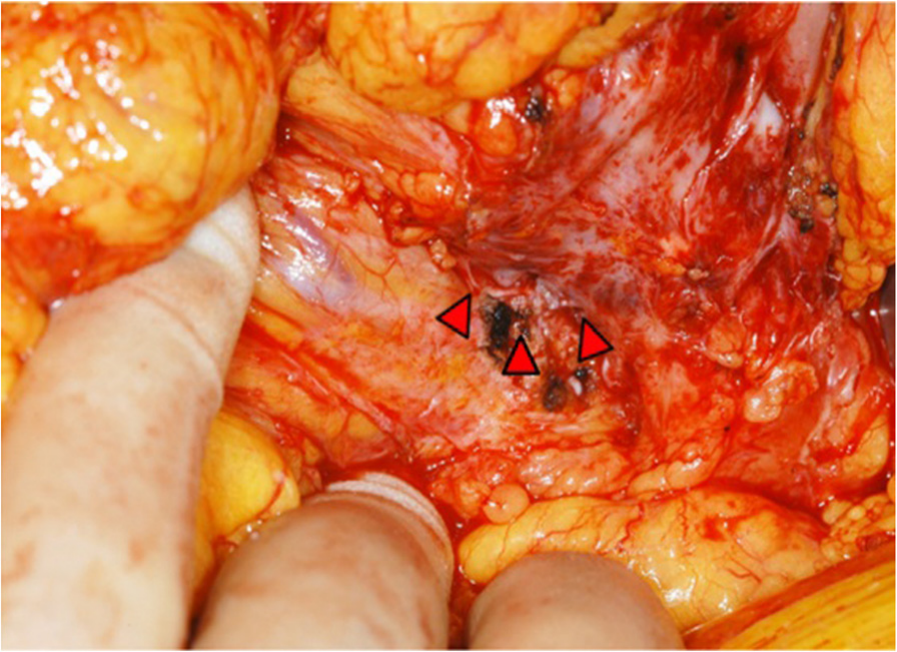
Intraoperative findings. The surrounding white connective tissue samples were found to be positive for pancreatic adenocarcinoma on pathological examination during surgery.

**Figure 5:**
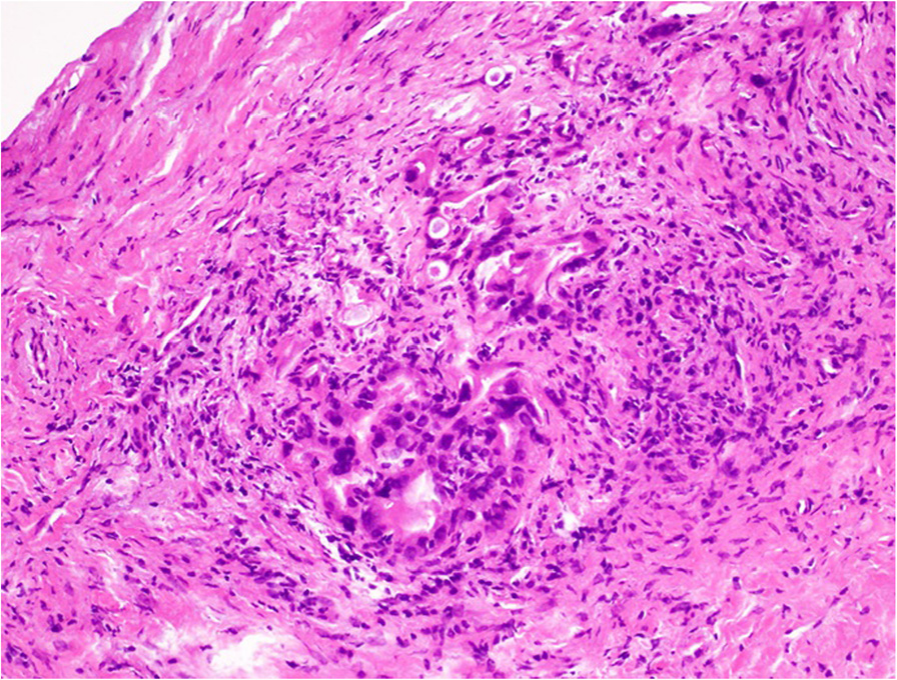
Pathological findings. The pathological findings showed invasive adenocarcinoma with an irregular small lumen and a small alveolar configuration (×20).

A 63-year-old woman underwent investigations for epigastric abdominal pain. She had no history of alcohol consumption or pancreatitis. Abdominal ultrasonography showed a moderate-sized pancreatic cyst on the pancreatic body with extrapancreatic growth. An enhanced computed tomography scan (Toshiba Aquilion™ 64) revealed that the massive simple cyst in the pancreatic body was an extrapancreatic growth with poor enhancement, which was approximately 52 × 32 × 23 mm in size. The cyst had developed extrapancreatically and the main pancreatic duct had slight dilatation with compression of the pancreatic parenchyma. There was neither a mass lesion nor a low-density area suggestive of pancreatic cancer. The pancreatic parenchyma was compressed around the cyst. We did not suspect pancreatic cancer based on CT imaging alone (Figure 1). The simple cyst in the pancreatic body showed low signal intensity on T1-weighted MRI and high intensity on T2-weighted MRI. Additionally, MRI (Siemens Magnetom Aera 1.5 T) showed slight dilatation of the main pancreatic duct. The compressed pancreatic parenchyma remained unchanged around the cyst on MRI (Figure 2-A, B). MRCP findings are shown in Figure 2-C. Endoscopic ultrasonography showed a 40-mm cystic lesion with a thick wall and an intramural nodule in the cyst lesion. There was no mass lesion in the pancreatic parenchyma. Endoscopic retrograde cholangiopancreatography (ERCP) revealed no mucinous discharge and no enlargement of the ampulla of Vater. ERCP also showed extrinsic compression of the main pancreatic duct from the pancreatic head to the body. Washing cytology from the pancreatic duct was classified as Class III. Blood tests for pancreatic enzymes (amylase, lipase, and elastase) and serum levels of tumor markers (CEA, CA19-9, DUPAN-2, and Span-1) were all within the normal range. The differential diagnoses were mucinous cyst neoplasm (MCN), serous cyst neoplasm (SCN), intraductal papillary mucinous neoplasm (IPMN), pseudocyst with chronic pancreatitis, and pancreatic ductal carcinoma, but we were unable to reach a definitive diagnosis preoperatively. We had planned a pancreaticoduodenectomy but we could not remove the tumor because of significant invasion of the surrounding tissue (portal vein, superior mesenteric vein, etc.). The fluid content of the cyst was serous and not mucinous. Aspiration cytology from the pancreatic cyst revealed papillary cell clumps with nuclear expansion and clear nuclear bodies. The cells had irregular nuclei with substantial chromatin content. However, these cells were few and the findings were therefore classified as Class III (no malignancy) (Figure 3). The surrounding white connective tissue samples were found to be positive for pancreatic adenocarcinoma on pathological examination during surgery (Figure 4). Histopathology is shown in Figure 5. The pathological findings demonstrated invasive adenocarcinoma with an irregular small lumen and small alveolar configuration. We initiated chemoradiotherapy after the operation.

## Discussion

This case of pancreatic cancer with a moderate-sized cyst developing into an extrapancreatic growth is extremely rare and has not previously been reported. In pancreatic ductal carcinoma, retention cysts may occur secondary to intraluminal obstruction of the pancreatic ducts. Nitta et al. [1] described a series of cystic pancreatic ductal adenocarcinomas that had distinctive morphologic and immunohistochemical features. In that report, the maximal cyst diameter was 2.0 cm or more in all patients (range, 2.0–5.0 cm; mean, 3.7 cm). The average tumor size including the multiple large cystic structures was 6.0 cm (range, 4.0–8.0 cm) [1]. Cystic tumors of the pancreas occur with less frequency than solid lesions, and are often detected incidentally, as many of these lesions are small and asymptomatic [2]. Cystic lesions of the pancreas can be classified into three groups: pseudocysts, pancreatic cystic tumors, and true cysts. Pseudocysts occur following acute pancreatitis or acute exacerbation of chronic pancreatitis; pancreatic carcinoma; and abdominal trauma. Pancreatic cystic tumors consist of three types: serous tumors, mucinous tumors, and solid pseudopapillary tumors. For pancreatic cysts, the aim of imaging is to differentiate cystic tumors from tumor-like lesions, to further characterize the cystic tumors, and to distinguish benign tumors (which do not usually require surgical excision) from borderline or malignant tumors that must be resected whenever possible [3-7].

Kosmahl et al. [8] reported that a considerable number of pancreatic ductal adenocarcinomas and their variants display cystic features and must therefore be considered in the differential diagnosis of cystic neoplasms of the pancreas. Moreover, not all of the cystic structures they observed were neoplastic in nature. They may also represent nonneoplastic changes, such as retention cysts and inflammatory pseudocysts. Other report showed that most of the cystic pancreatic ductal adenocarcinomas reported in the literature were poorly differentiated tumors with pseudocystic changes [9-11]. In our case, the content of the cyst was nonmalignant and the cyst was a simple pseudocyst. We could not determine the histological cancer type because we could not obtain an adequate sample from the pancreatic cancer.

In our case, the patient had no history of pancreatitis. Of course, we suspected pancreatic ductal carcinoma with presence of a pancreatic cyst, but there was neither a mass lesion nor a low-density area suggestive of pancreatic cancer. We did not immediately suspect pancreatic cancer at first, as the development of a moderate-sized cyst with extrapancreatic growth is extremely rare and the levels of known tumor markers were not elevated. Therefore, we initially suspected that a massive benign cyst (MCN, SCN, or IPMN) resulted in stenosis of the main pancreatic duct. We were unable to reach a definitive diagnosis prior to the operation. We planned a pancreaticoduodenectomy to reach a definitive diagnosis; however, we could not remove the tumor because of significant invasion of the surrounding tissue. The fluid content of the cyst was serous and aspiration cytology from the pancreatic cyst was deemed to be Class III (no malignancy), but the surrounding white connective tissue samples were found to be positive for pancreatic adenocarcinoma on pathological examination during surgery. In this case, the content of the cyst was not malignant and the cyst was a simple pseudocyst. We repeated imaging (CT, MRI, endoscopic ultrasound, etc.) once more after the operation, and there were no mass lesions or low-density area suggestive of pancreatic cancer. In retrospect, we think that the slight pancreatic duct dilation was the only finding suggestive of pancreatic cancer.

If a pancreatic cyst is found in patients who have normal tumor marker levels and no history of pancreatitis, we should always consider the possibility of pancreatic cancer. Slight pancreatic duct dilation may be a diagnostic clue in these cases. In the end, we could not resect the tumor because it was too advanced. However, regardless of whether the tumor is resectable, we need to identify these cases because they strongly influence disease prognosis.

In the present case, we could not conduct an in-depth pathological examination for pancreatic cancer because we could not obtain an adequate sample from the pancreatic cancer. However, we had the impression that this tumor was more invasive than the usual invasive ductal carcinoma.

Despite the development and widespread use of imaging techniques, it remains difficult to preoperatively establish an accurate diagnosis of pancreatic cancer that is accompanied by moderate-sized pancreatic cystic lesions. An accurate diagnosis requires careful consideration of the complete medical history of the patient in addition to the appropriate use of imaging and endoscopic approaches. It is important to carefully observe changes of the pancreatic parenchyma around massive cysts, regardless of their size. If a pancreatic cyst with slight pancreatic duct dilation is found in patients who have normal tumor marker levels or no history of pancreatitis and changes of the pancreatic parenchyma that are suggestive of pancreatic cancer are not observed, we should always consider the possibility of pancreatic cancer. Slight pancreatic duct dilation may be a diagnostic clue in these cases.

## Conclusions

It is difficult to diagnose pancreatic cancer with pseudocyst preoperatively. If a pancreatic cyst is found in patients who have normal tumor marker levels or no history of pancreatitis, we should always consider the possibility of pancreatic cancer. In such cases, slight pancreatic duct dilation may be a diagnostic clue.

## Consent

Informed consent was obtained from the patient for publication of this case.

## Abbreviations

CT: Computed tomography
MRI: Magnetic resonance imaging
ERCP: Endoscopic retrograde cholangiopancreatography
MCN: Mucinous cyst neoplasm
SCN: Serous cyst neoplasm
IPMN: Intraductal papillary mucinous neoplasm
